# Does the Prediction Accuracy of Osteoporotic Fractures by BMD and Clinical Risk Factors Vary With Fracture Site?

**DOI:** 10.1002/jbm4.10238

**Published:** 2019-10-29

**Authors:** L Iconaru, M Moreau, V Kinnard, F Baleanu, M Paesmans, R Karmali, JJ Body, P Bergmann

**Affiliations:** ^1^ Department of Endocrinology CHU Brugmann, Université Libre de Bruxelles Brussels Belgium; ^2^ Data Centre Institut Jules Bordet, Université Libre de Bruxelles Brussels Belgium; ^3^ Department of Internal Medicine, CHU Brugmann Université Libre de Bruxelles Brussels Belgium; ^4^ Department of Nuclear Medicine CHU Brugmann, Université Libre de Bruxelles Brussels Belgium

**Keywords:** BMD, FRACTURE SITE, FRAX, OSTEOPOROSIS, RISK FACTORS

## Abstract

Several clinical risk factors (CRFs) have been shown to predict the risk of fragility fractures independently of BMD, but their accuracy in the prediction of a particular fracture site has not been extensively studied. In this study based on longitudinal data from the FRISBEE cohort (Fracture Risk Brussels Epidemiological Enquiry), we evaluated if CRFs are specific for sites of incident osteoporotic fractures during follow‐up. We recruited 3560 postmenopausal women, aged 60 to 85 years, from 2007 to 2013, and surveyed yearly for the occurrence of fragility fractures during 6.2 years (median). We analyzed the association between CRFs included in the FRAX (fracture risk assessment tool) model or additional CRFs (falls, sedentary lifestyle, early untreated menopause, diabetes, use of selective serotonin reuptake inhibitors or proton pump inhibitors) and the first incident validated major osteoporotic fracture (MOF; *n* = 362; vertebra, hip, shoulder, and wrist) or other major fractures (*n* = 74; ankle, pelvis/sacrum, elbow, knee, long bones). Uni‐ and multivariate analyses using the Cox proportional hazards model were used. For MOFs considered together, the risk of fracture was highly associated in uni‐ and multivariate analyses (*p*<0.01) with osteoporosis (*T*‐score < −2.5), prior fracture, age, BMD (assessed by DXA), and fall history (HR 2.34, 1.82,1.71, 1.38, and 1.32, respectively). For each site analyzed separately, prior OF, age, smoking, and total hip BMD remained independent predictors for hip fractures (HR 5.72, 3.98, 3.10, 2.32, and 1.92, respectively); osteoporosis, age, prior OF, glucocorticoids, and spine BMD for vertebral fracture (HR 2.08, 1.87, 1.78, 1.76, and 1.45, respectively); osteoporosis, prior OF, and femoral neck BMD (HR 1.83, 1.60, and 1.56, respectively) for wrist fracture; osteoporosis, prior OF, and spine BMD (HR 2.48, 1.78, and 1.31, respectively) for shoulder fracture; prior OF and diabetes (HR 2.62 and 2.03) for other major fractures. Thus, a prior fracture and BMD were the best predictors of fracture risk at any site. Other CRFs have a weaker predictive value, which is a function of the site of a future fracture. © 2019 The Authors. *JBMR Plus* published by Wiley Periodicals, Inc. on behalf of American Society for Bone and Mineral Research.

## Introduction

Osteoporotic fractures constitute a major public health concern worldwide because a fragility fracture leads to higher disability and increases the risk for new fractures.^1^, ^2^ Fracture events have a substantial impact in terms of quality of life, particularly after hip and vertebral fracture.^3^ Population attributable risk for mortality from fracture is similar to that from cardiovascular disease and diabetes, highlighting their importance and potential benefit for early prevention treatment (population mortality attributable to any fracture without comorbidity is 9.2% in women, which is similar in magnitude to other well‐described causes of mortality, including cardiovascular disease, diabetes, and cancer).^4^

Deaths after a fracture are in part related to comorbidities, but can also result from the fracture event itself, either directly or indirectly. Hip fractures are the most relevant fractures in terms of mortality, worse quality of life, functional dependence, and social and economic costs, especially among the elderly.^5^ Besides hip and vertebral fractures that are associated, respectively, with a threefold and 2.7‐fold increase in mortality, pelvis, humerus, clavicle, proximal tibia/fibula, elbow, distal forearm, and ribs fractures are also associated with mortality HRs ranging from 1.3 to 3.4.^4^

Because osteoporotic fractures cause a huge financial burden worldwide, osteoporosis should ideally be prevented, diagnosed, and treated before a fracture occurs. As a low BMD is an important factor predicting increased fracture risk, the diagnosis of osteoporosis relies on the assessment of BMD by DXA.^6^ Although low BMD at any skeletal site can predict osteoporotic fracture, site‐specific measurements are generally better for their respective sites.^7^ A low BMD is also a rather strong predictor of mortality: for each SD decrease in BMD, the mortality risk is increased by approximately 1.5‐fold.^8^

However, to foresee who is most at risk of fracture remains a challenge. Osteoporosis, defined by a *T*‐score < −2.5 SD by DXA at the hip, femoral neck, or lumbar spine, is a major contributor to fracture risk, but there is a continuous inverse relationship between BMD and fracture risk. At least 50% of fractures occur in persons with a normal or osteopenic BMD.

Several other clinical factors have been shown to be independent contributors to the risk of fracture; taking them into account improves the identification of patients at high risk of fracture.^9^ These factors reflect contributors to fracture other than bone mass, such as the so‐called bone quality, a concept including bone microarchitecture and the strength of bone material.

Some of these clinical factors have been integrated into models that allow the estimation of fracture risk at 5 to 10 years. The most popular of them is the fracture risk assessment tool (FRAX) with or without BMD. The FRAX tool estimates an individual's probability of hip fracture or a major osteoporosis‐related fracture (MOFs: hip, clinical spine, shoulder, or wrist) in the next 10 years. The risk factors included in the model besides BMD are age, sex, a prior fragility fracture, a parental history of hip fracture, current smoking, the use of systemic corticosteroids, excess alcohol intake, low BMI, rheumatoid arthritis (RA), and several causes of secondary osteoporosis.

Specific studies and meta‐analyses showed that several other factors, not included in the FRAX tool, are independent contributors to the risk of fracture. They include the risk of falls,^10^, ^11^ early untreated menopause,^12^, ^13^ the quality of sleep, and diabetes.^14^, ^15^ The use of psychoactive drugs and proton pump inhibitors (PPIs) has also been shown to be a risk factor for fractures in some studies.^16^, ^17^

If several clinical risk factors (CRFs) have been shown to predict the risk of osteoporotic fractures independently of BMD, the accuracy of the prediction for a particular site has not been extensively studied. The site of an osteoporotic fracture will determine the burden of the associated mortality and morbidity; it is important to determine if risk factors for osteoporotic fractures have similar predictive values on the occurrence of a fracture whatever the fracture site.

The FRISBEE study (the Fracture Risk Brussels Epidemiological Enquiry) aims at validating and integrating several independent CRFs to develop a fracture risk model in a well‐characterized patient population studied prospectively. Here, we took advantage of this cohort to evaluate if risk factors for osteoporotic fractures vary according to the site of the first incident validated fracture during follow‐up.

## Materials and Methods

The FRISBEE cohort consists of 3560 postmenopausal women recruited between 2007 and 2013 who are surveyed yearly for the occurrence of fragility fractures. The study design has been detailed and reported previously.^18^

We conducted separate analyses for women with one of the four major osteoporotic fractures (MOFs: vertebra, hip, shoulder, and wrist), and other major fractures (defined as fractures at the following sites: ankle, pelvis and sacrum, elbow, knee (except patella), distal humerus, proximal forearm, lower leg, distal femur). Other fractures, considered as minor, were not considered in this study. We considered only the first incident fracture after inclusion, nontraumatic and validated by radiological or surgical reports, and declared and undeclared fractures (found in the medical files). We compared the predictive values of risk factors at baseline, included or not in the FRAX model, according to the fracture site.

Besides CRFs included in the FRAX model and BMD values measured at the lumbar spine, the femoral neck, or the total hip, several additional risk factors not included in the FRAX model were also registered. We selected these additional CRFs after a systematic review of recent cohorts and studies, using the following criteria: a population‐based prospective study that aimed to determine an association between diverse CRFs and fracture risk, which included at least 1000 women and was published in English. We retained six of these, in which at least three CRFs used for the calculation of the FRAX score and one or more CRFs not included in the FRAX model were taken into consideration.^18^ They included early non‐substituted menopause (occurring before the age 45 years) considered as a cause of secondary osteoporosis in the FRAX® score^19^, ^20^ but that we separately considered in this analysis: fall history (documented using frequency of falls),^20^, ^21^ sedentary lifestyle (i.e. the lowest activity level evaluated according to the 6‐level scale, adapted from the International Physical Activity Questionnaire (IPAQ) World Health Organization score) and diabetes type 2.^20^, ^22^ We did not perform a separate analysis for patients with type 1 diabetes because of their small number. The information about PPI and selective serotonin reuptake inhibitor (SSRI) use was available in only 2093 and 723 women, respectively.

Any fracture reported by the study participants was carefully validated by obtaining written radiological and/or surgical reports. We also included fractures not reported by study participants, but validated by such reports.

We analyzed the association between the site of the first incident validated fracture and every assessed CRF, included or not in the FRAX model.

### Statistical analyses

Uni‐ and multivariate analyses using the Cox proportional hazards model were used. In the multivariate analyses, a backward selection method of the covariates was used. Factors tested for possible inclusion in the multivariate models were selected as those found with a *p* value <0.3 in the univariate analysis. The proportional hazards assumption was verified on all the variables using cumulative sums of Martingale residuals (Assess Statement and Resample option in the SAS procedure, Proc Phreg). For multivariate analyses, BMD and age were modeled as continuous variables. For each endpoint, two models were created (“BMD” and “Osteoporosis”): one used the variable “osteoporosis” and the other the three BMD values. In the BMD model, HRs were estimated for a decrease of 0.100 g/cm^2^ of BMD. Osteoporosis therapy was considered as a covariate.

All significance probabilities were for two‐sided tested. We set the threshold for significance at *p* < 0.05 without multiplicity adjustment.

## Results

The median age of the 3560 women at inclusion in the FRISBEE study was 70 years. The most frequently encountered CRFs included in the FRAX model were a prior fragility fracture (29%), a parental history of hip fracture (13%), causes of secondary osteoporosis (14%) including early nonsubstituted menopause (5.7%), a BMI <20 kg/m2 (8.3%), excessive alcohol intake (7.7%), and use of corticosteroids (7.7%). The prevalence of CRFs not considered in the FRAX model was rather high at inclusion in our population of volunteer women: 703 women (19.8%) had a history of fall(s) in the last 6 months, 198 (5.6%) had a sedentary lifestyle, and 251(7%) had diabetes (type 1 = 9, type 2 = 242). In the subgroups for whom the information on the use of PPIs and SSRIs was available, the percentage of users was rather high: 24.9% and 29.5%, respectively. Only 12.8% of the patients were treated for osteoporosis at inclusion.

Between 2007 and 2018, 953 fractures occurred (median follow‐up 6.2 years; range 0.36 to 11.1 years). The first incident MOF (*n* = 436) occurred at the hip (52 patients), spine (120 patients), wrist (122 patients), and shoulder (68 patients). In 74 patients, the first other major fracture occurred at another site.

**Figure 1:**
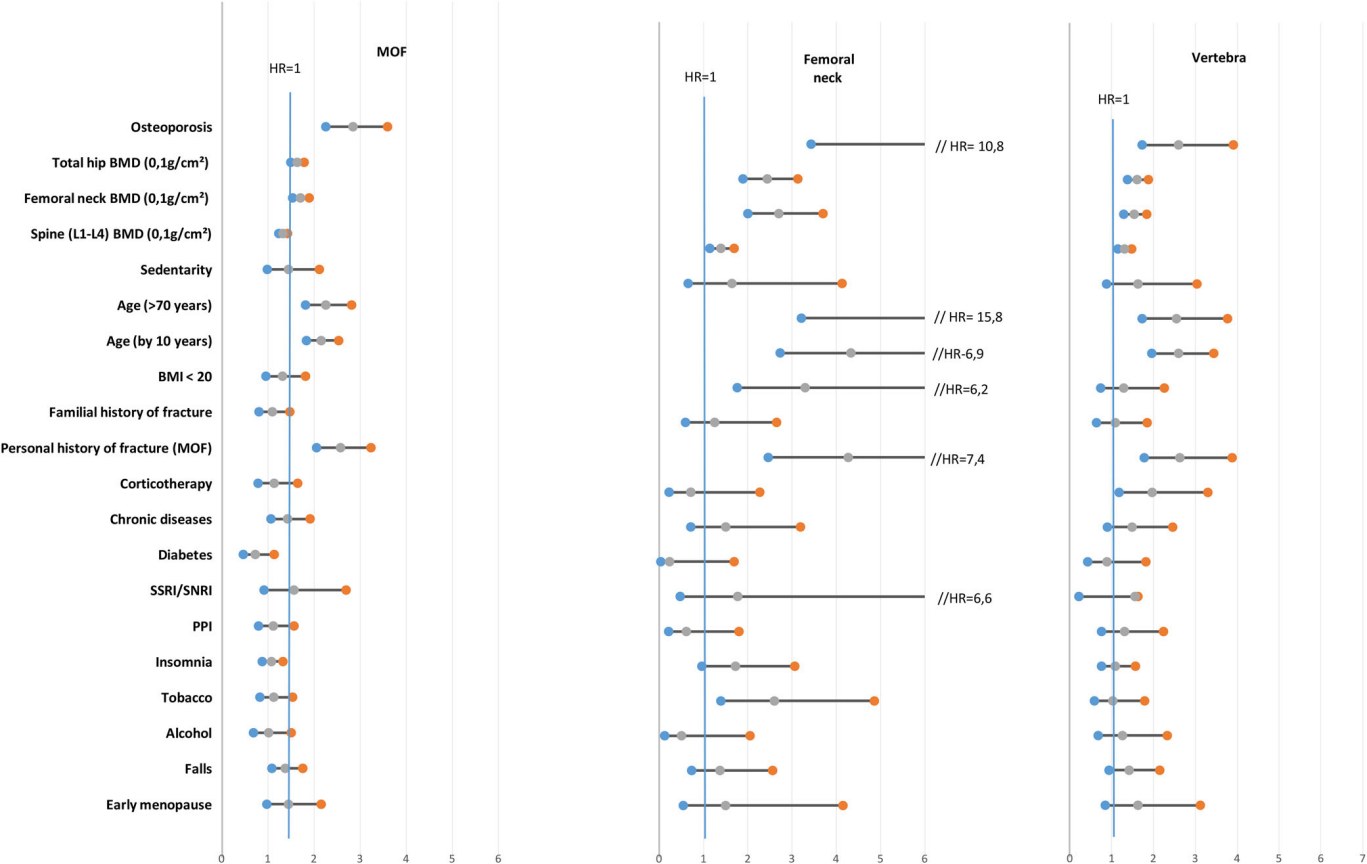
Univariate adjusted hazard ratios for fracture risk factors according to the site of the first incident fracture (major osteoporotic fracture, hip and vertebra). MOF = major osteoporotic fracture; PPI = Proton Pump Inhibitors; SSRI = selective serotonin reuptake inhibitor; SNRI = serotonin‐norepinephrine reuptake inhibitor.

The predictive value of CRFs for MOFs occurrence taken all together is shown in Table 1 and Fig. 1. BMD at any site, osteoporosis, age, and a history of fragility fracture before study inclusion were highly significantly associated with the occurrence of an incident fracture (*p* < 0.0001), with the higher risk gradient for osteoporosis, a prior fragility fracture, and age (HR 2.84, 2.56, and 2.26, respectively) in univariate analysis. A weaker association was observed for a fall history, sedentary lifestyle, and early menopause (Table 1, left panel and Fig. 1, left panel). In multivariate analysis, only hip BMD, osteoporosis, age, and a prior fragility fracture remained significantly associated with an incident MOF. A weaker association persisted between the occurrence of MOFs and fall history and spine BMD. HRs were generally slightly higher in the osteoporosis model (Table 1, right panel).

**Table 1:** Univariate and Multivariate Analyses for Clinical Risk Factors for Major Osteoporotic Fractures Considered Together

	Major osteoporotic fracture				
	Univariate analysis		Multivariate analyses		
	HR (CI)	*p* value	HR (CI) (osteoporosis model)	HR (CI) (BMD model)	*p* value
Osteoporosis	2.84 (2.25–3.59)	<0.0001	2.34 (1.84–2.98)		<0.0001
Neck BMD (0.1 g/cm^2^)	1.70 (1.53–1.89)	<0.0001			
Hip BMD (0.1 g/cm^2^)	1.63 (1.49–1.78)	<0.0001		1.38 (1.25–1.56)	<0.0001
Spine BMD (0.1 g/cm^2^)	1.32 (1.23–1.42)	<0.0001		1.11 (1.01–1.20)	0.033
Sedentary lifestyle	1.44 (0.98–2.11)	0.06			
Age (>70 years)	2.26 (1.81–2.81)	<0.0001	1.85 (1.47–2.32)	1.71 (1.35–2.16)	<0.0001
BMI < 20 kg/m^2^	1.31 (0.95–1.81)	0.10			
Familial history of fractures	1.08 (0.80–1.47)	0.62			
Prior fragility fracture	2.56 (2.05–3.23)	<0.0001	1.97 (1.55–2.50)	1.82 (1.43–2.33)	<0.0001
Glucocorticoid therapy	1.13 (0.78–1.64)	0.52			
Rheumatoid arthritis	1.40 (0.58–3.4)	0.45			
Diabetes	0.73 (0.46–1.13)	0.16			
PPI	1.11 (0.79–1.56)	0.56			
SSRI/SNRI	1.56 (0.91–2.69)	0.11			
Sleep disturbances	1.07 (0.87–1.32)	0.54			
Smoking	1.12 (0.82–1.53)	0.47			
Alcohol	1.01 (0.68–1.5)	0.95			
Fall history	1.38 (1.08–1.75)	0.0098	1.28 (1.00–1.64)	1.32 (1.03–1.69)	0.029/0.049
Early menopause	1.44 (0.97–2.15)	0.07			

PPI = Proton Pump Inhibitors; SSRI = selective serotonin reuptake inhibitor; SNRI = serotonin‐norepinephrine reuptake inhibitor.

**Figure 2:**
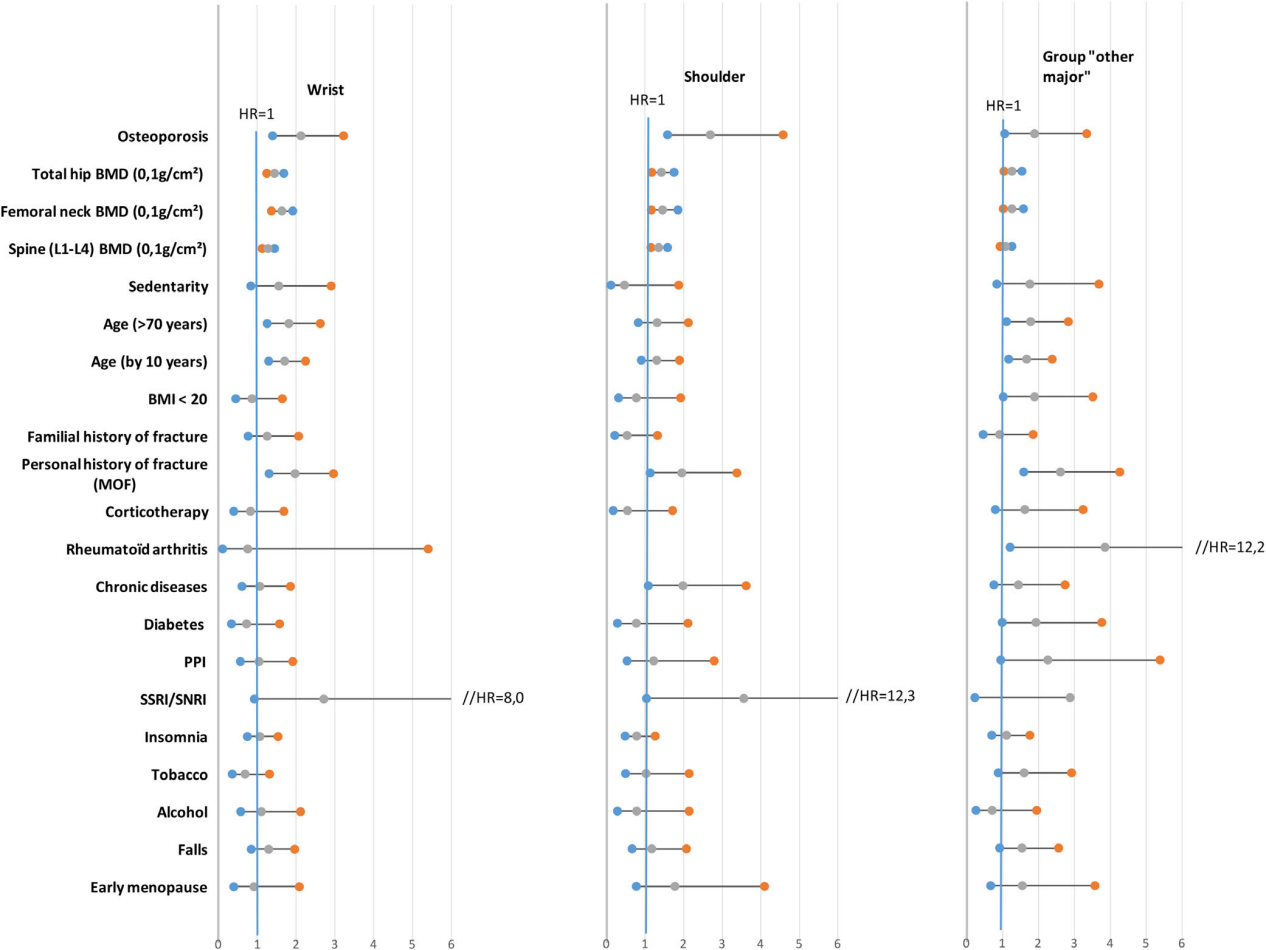
Univariate adjusted hazard ratios for fracture risk factors according to the site of the first incident fracture (wrist, shoulder, and other major fractures). MOF = major osteoporotic fracture; PPI = Proton Pump Inhibitors; SSRI = selective serotonin reuptake inhibitor; SNRI = serotonin‐norepinephrine reuptake inhibitor.

The predictive value of CRFs on specific fracture sites is shown in Figs. 1 and 2, and Table 2. In univariate analysis, BMD, osteoporosis, and a prior fragility fracture were all predictive of any MOF and of other major osteoporotic fractures, with a higher risk gradient for the hip, except for spine BMD. A low BMI and smoking habit were only associated with hip fracture, RA with vertebral fractures and other major fragility fractures, glucocorticoids with vertebral fractures, and SSRI use only with wrist fracture. Diabetes type 2 was only associated with other major fractures.

**Table 2:** Multivariate Adjusted Hazard Ratios and Confidence Intervals for Clinical Risk Factors According to the Site of the First Incident Fracture (Only Factors With a Highly Significant Level (*p* < 0.01) of Association With the Fracture Site Are Shown)

	Hip (*n* = 52)	Vertebral (*n* = 120)	Wrist (*n* = 122)	Shoulder (*n* = 68)	Other major (*n* = 74)
Osteoporosis	3.98 (2.22–7.15)	2.08 (1.37–3.16)	1.83 (1.18–2.78)	2.48 (1.45–4.26)	
Neck BMD (0.1 g/cm^2^)			1.56 (1.31–1.88)		
Total hip BMD (0.1 g/cm^2^)	1.92 (1.49–2.50)				
Spine BMD (0.1 g/cm^2^)		1.45 (1.23–1.69)		1.31 (1.11–1.56)	
Age (>70 years)					
Osteoporosis model	6.36 (2.65–15.4)	2.16 (1.44–3.24)			
BMD model	5.72 (2.35–13.9)	1.87 (1.24–2.83)			
Prior fragility fracture					
Osteoporosis model	2.67 (1.47–4.82)	1.94 (1.29–2.91)	1.60 (1.05–2.43)	1.75 (1.00–3.06)	2.62 (0.60–4.30)
BMD model	2.32 (1.27–4.22)	1.78 (1.18–2.68)	1.67 (1.10–2.54)	1.78 (1.00–3.06)	2.62 (1.60–4.30)
Glucocorticoid therapy					
Osteoporosis model		1.72 (1.00–2.96)			
BMD model		1.76 (1.02–3.02)			
Rheumatoid arthritis					
Osteoporosis model					3.73 (1.17–11.8)
BMD model					3.73 (1.17–11.8)
Diabetes					
Osteoporosis model					2.03 (1.04–3.96)
BMD model					2.03 (1.04–3.96)
Smoking					
Osteoporosis model	3.20 (1.62–6.36)				
BMD model	3.10 (1.55–6.21)				

In a multivariate analysis (Table 2), only total hip BMD, a diagnosis of osteoporosis, age, a prior fragility factor, and smoking remained independent predictors of hip fractures; spine BMD, a diagnosis of osteoporosis, age, a prior fragility factor, and glucocorticoid therapy remained associated with spine fracture. A wrist fracture was better predicted by femoral neck BMD, a diagnosis of osteoporosis, and a prior fragility fracture; a shoulder fracture was better predicted by spine BMD, a diagnosis of osteoporosis, and a prior fragility fracture. For other major fractures, only a prior fragility fracture and type 2 diabetes remained independent predictors.

## Discussion

Several CRFs predicting osteoporotic fractures have been described, but the association between these risk factors and a particular site of incident fracture has not been studied extensively.

Also, the FRAX algorithm has some limitations, which may result in over‐ or underestimation of fracture risk in an individual patient. One of these is that it includes dichotomous (yes or no) input for clinical risk factors that are associated with variable risk depending on dose and duration of exposure (eg, number of fractures, glucocorticoid therapy, cigarette smoking, and alcohol intake). Besides, the algorithm does not take into consideration all risk factors (eg, falls, bone turnover, gait, spine BMD, diabetes, …); it may thus underestimate fracture probability in some individuals, for example, with multiple or recent fractures, lumbar spine BMD much lower than femoral neck BMD, high‐dose glucocorticoid exposure, a parental history of nonhip fragility fracture, or diabetes mellitus.^23^, ^24^, ^25^

In this study, as shown previously, we found that the risk of a MOF at any site is highly associated, in uni‐ and multivariate analyses, with BMD measured at any site, a diagnosis of osteoporosis according to the WHO definition (*T*‐score < −2.5 DS by DXA at the spine, total hip, or femoral neck), age, a prior fracture, and fall history. However, a parental history of hip fracture, smoking, excessive alcohol consumption, RA, treatment with glucocorticoids, and a low BMI, all classical risk factors included in the FRAX model, were only weakly and not significantly associated with the risk of MOF. The measurement of BMD at any site predicts fracture risk for MOFs equally well. BMD measured at any site, osteoporosis, and a prior fracture remain significantly associated with incident fracture at any site. However, when analyzed site by site, the relation with age, which was highly significant in the multivariate analysis for incident MOFs, remained significant only for spine and hip, with a HR for hip of 5.7 for patients older than 70, at any value of BMD. Several epidemiological studies,^26^ but not all,^27^ have shown that, although hip and vertebral fracture incidence continue to rise steeply with advancing age, that of wrist and other peripheral fractures tend to plateau between 60 and 70. An explanation could be that, because of their better neuromuscular control, younger people are more able to protect themselves with their arms and legs and therefore sustain more distal than proximal fractures.^28^

The strength of the association with BMD measured at different sites and fracture risk was a function of the fracture site. As shown previously, total hip BMD and femoral neck BMD were more predictive of hip fracture, with a HR of 1.92 per SD decrease. For other MOFs, the HR was lower (1.3 to 1.6), independently of the site of BMD measurement. It was the lowest for other major fractures, with a HR of only 1.1 for spine BMD. The association with BMD remained significant in multivariate analysis for total hip BMD and hip fracture, spine BMD and vertebral fracture, femoral neck BMD and wrist fracture, spine BMD and shoulder fracture, but not for other major fractures for which the most important CRF was a prior fragility fracture, with a HR of 2.62 in multivariate analysis. As expected, the same was true for a diagnosis of osteoporosis, which did not emerge as a significant risk factor for such fractures in multivariate analysis.

A fall history was significantly associated with the risk of fracture in both univariate and multivariate analyses, showing the importance to assess the risk of falling and of interventions aimed to reduce this risk. This assessment should be incorporated into routine clinical osteoporosis care.

Some risk factors were only associated with specific fracture sites. A low BMI and smoking habit were only associated with hip fractures, whereas treatment with glucocorticoids was an independent risk factor for spine fractures only.^29^, ^30^ For glucocorticoids use, we considered all patients receiving ≥5 mg prednisolone daily or its equivalent for a duration of ≥3 months, but we did not perform analyses taking into account the glucocorticoid dose because of insufficient data. RA was associated with vertebral fractures and other major fractures, but only in univariate analysis. This association could be explained by local bone loss in the vicinity of affected joints. Type 2 diabetes emerged as another independent risk factor for such fractures only in univariate analysis, an observation consistent with the increased number of peripheral fractures that has been described in patients with diabetes.^15^ Psychoactive drugs increased the risk of shoulder fracture, probably through their effect on the risk of fall resulting from sedation, increased reaction time, and disrupted balance and gait. However, the association with psychoactive drugs disappeared after correcting for age and BMD in multivariate analysis.

Adding treatment for osteoporosis at inclusion as a covariate in the multivariate analysis did not change the results.

In several studies in women, a number of bone turnover markers (BTMs) were associated with an increase in fracture risk and the predictive ability of an increase in BTMs was independent of BMD. More recently, other biological, nonclassical bone markers, such as a peptide derived from periostin, were shown to be predictive of fracture risk and to improve fracture prediction when introduced in FRAX or added to BMD.^31^

Thus, BTMs hold promise as an independent predictor for fracture and could improve the identification of women with the highest rate of bone loss and osteoporosis risk. However, as discussed in a recent review,^32^ the results of the studies lack consistency, in terms of the markers studied and endpoints, and there is a need for carefully conducted studies before they can be used in clinical practice with the purpose of fracture prediction. In our study, BTM measurements were not available.

A more recent publication suggests that fracture risk assessment should consider regional factors in addition to classical risk factors, such as BMD.^33^

Our study has strengths and limitations. The major strengths reside in the prospective nature of our cohort study with systematic annual interviews and validation by radiological and/or surgical reports of all considered fractures. In patients with multiple fractures, we decided to take into account only the first incident fracture. It could be argued that we should have considered all first fractures in each group. The first option was chosen because, at least in those who did not have a fracture at inclusion, this event changes the risk evaluation. The main limitation is the relatively small number of fractures, which could explain the absence of significant relationship between some CRFs and incident fractures, particularly when the sites were analyzed separately. This could explain why some CRFs emerging for MOFs as a group were not significant for each site. Lastly, our study only considers female subjects and the conclusions might thus not be valid for men with osteoporosis. Nevertheless, our data indicate that the predictive value of age or BMD values varies according to the fracture site.

In conclusion, this study confirms that the most universal predictors of any future fracture are a prior fracture, followed by BMD and a diagnosis of osteoporosis (*T*‐score < −2.5). Patients older than 70 years of age, particularly if they smoke, and if their BMI is low, are more at risk for hip fractures, independently of BMD. Treatment with glucocorticoids increases mainly the risk of spine fractures, whereas diabetes increases that of peripheral fractures. A fall history was also an important predictor of MOFs as a group.

Thus, this study reinforces the importance of a first fracture in finding those patients who are most at risk of new fractures and who should be imperatively enrolled for diagnosis and treatment of osteoporosis. Other CRFs have a weaker predictive value that is a function of the site of a future fracture.

Starting from the relationship between some CRFs and the specific site of fracture, there could be an opportunity for observational studies on the possible site‐specific antifracture effects of bone active drugs because the magnitude of the effect is affected by background fracture risk.^33^

## Disclosures

Authors declare no conflict of interest and no funding.
